# Data set on volatile compound of coffee flowers at different annual rainfall

**DOI:** 10.1016/j.dib.2019.104418

**Published:** 2019-08-21

**Authors:** Tati Suryati Syamsudin, Hafsah Hafsah, Iriawati Iriawati

**Affiliations:** School of Life Sciences and Technology, Institut Teknologi Bandung, West Java Indonesia

**Keywords:** Altitude, GC-MS, Metabolite profiling, Microclimate, SPME

## Abstract

This data informs about the profile of volatile compound of coffee flower (Coffee arabica) from different locations with different annual rainfall by using gas chromatography - mass spectrometry (GC-MS). The volatile compounds were captured by solid phase micro extraction (SPME) methods. The extract then subjected to GC-MS for separation and identification of compounds. The profile of volatile compound was provided in, Table 1, Table 2, Table 3 and Table 4.

**Table:** Specifications Table

Subject area	*Agriculture and Biological Science*
More specific subject area	*Biochemical diversity*
Type of data	*Table*
How data was acquired	*Volatile compound of Coffeaarabica flowers from four location were analyzed using gas chromatography (GC: 7890A, Agilent Technologies, Inc.) coupled with mass spectrometry (MS: 5975C, Agilent Technologies, Inc.).*
Data format	*Data (in Excel Worksheet)*
Experimental factors	*Flower samples are taken from four locations at the altitude higher than 1100 m above sea level with different average annual rainfall (1500–2000, 2000–2500, 2500–3000, 3000–3500 mm/year)*
Experimental features	• Identification of volatile compound, determination of retention times and measurement of peak area • Volatile compounds of coffee flower were compared among four location at different annual rainfall
Data source location	• Sukamakmur, Bogor Regency, West Java (S06°’40′12.9″ E107°00′40.6″) 1170 above sea level • Pangalengan, Bandung Regency, West Java (S07°09′27.1″ E107°33′35.9″) 1266 above sea level • Tambi, Wonosobo Regency, Central Java (S07°’15′25.1″ E109°33′36.1″) 1497 above sea level • Kamojang, Bandung Regency, West Java (S07°08′47.2″ E107°30′30.6.”) 1504 above sea level
Data accessibility	*Data are available in this article*

**Table:** 

**Value of the data** • The data showed the profile of volatile compound of *Coffea arabica* flowers from four locations at different annual rainfall • The data will be important for pollination study related to diversity of insect visitors on emitted volatile compound by coffee flower which will contribute to fruit mass formation and coffee bean quality • The data will be useful to find out an insect attractant on coffee flower which will contribute on pest/insect control • The data can be used for modeling the effect of rainfall on the secretion of volatile compound on coffee flowers • The data are important for many stakeholders include farmer, policy makers, researchers, scholars and academics to mitigate climate change especially rainfall fluctuations on coffee production

## 1 Data

The data about volatile compound profile of coffee flowers from different locations at different annual rainfall was provided on the Microsoft Excel Worksheet (Table 1, Table 2, Table 3, Table 4). The data contain the retention times, names of volatile compound and the peak area of coffee flowers.

**Table 1:** Retention times, names of the identified compounds, and relative peak areas (%) in the GC chromatogram from coffee flower at 1500–2000 mm/year annual rainfall.

Retention Time (min.)			Compounds	Relative Peak Area (%)		
1	2	3		1	2	3
2.823	2.841		Cyclobutanol	0.32	0.29	n.d
3.233	3.233	3.245	Butanenitrile, 2-methyl-	3.64	3.85	3.43
4.303			1,3-Dioxolane, 2-ethenyl-4-methyl-	5.01	n.d	n.d
	4.315		2-Butenoic acid, butyl ester	n.d	3.47	n.d
7.924			Oxalic acid, pentyl propyl ester	0.87	n.d	n.d
11.266	11.272	11.280	β-myrcene	0.77	0.58	0.63
12.544	12.562	12.562	D-Limonene	0.32	0.25	0.22
12.866	12.854	12.859	Benzyl alcohol	0.35	0.39	0.46
	13.222	13.228	β-Ocimene	n.d	0.55	0.56
13.222			1,3,6-Octatriene, 3,7-dimethyl-, (Z)	0.57	n.d	n.d
14.043	14.043	14.049	Ethyl 2-(5-methyl-5-vinyltetrahydrofuran-2-yl)propan-2-yl carbonate	0.07	0.09	0.10
		14.560	Cyclohexene, 1-methyl-4-(1-methylethylidene)-	0.13	n.d	n.d
14.774	14.780		Benzoic acid, methyl ester	0.93	1.40	n.d
		14.828	3-Methyl-2-(2-methyl-2-butenyl)-furan	n.d	n.d	0.20
14.953			Geranyl benzoate	0.53	n.d	n.d
	14.965	14.958	Linalool	n.d	1.12	0.79
15.886	15.892	15.892	2,4,6-Octatriene, 2,6-dimethyl-, (E,Z)-	0.11	0.04	0.04
	17.301	17.301	(3R,6S)-2,2,6-Trimethyl-6-vinyltetrahydro-2H-pyran-3-ol	n.d	0.02	0.02
17.533	17.533		3,6-Octadienal, 3,7-dimethyl-	0.02	0.02	n.d
		17.539	Isogeranial	n.d	n.d	0.02
		17.812	Terpineol	n.d	n.d	0.02
17.902	17.902	17.902	Methyl salicylate	0.07	0.09	0.03
17.997	17.997	17.997	Dodecane	0.05	0.07	0.06
18.645	18.657	18.669	6-Octen-1-ol, 7-methyl-3-methylene	0.02	0.02	0.02
18.966	18.990	18.996	2,6-Octadien-1-ol, 3,7-dimethyl-,(Z)-	3.11	3.29	3.56
19.055		19.073	3,6-Octadien-1-ol, 3,7-dimethyl-, (Z)-	0.14	n.d	0.15
	19.067		Santolina triene	n.d	0.13	n.d
19.269	19.275	19.281	2,6-Octadienal, 3,7-dimethyl-, (Z)	0.22	0.18	0.17
19.697	19.721	19.745	2,6-Octadien-1-ol, 3,7-dimethyl-, (E)-	1.91	2.21	2.79
20.108	20.114	20.120	2,6-Octadienal, 3,7-dimethyl-, (E)	0.25	0.20	0.19
20.470			6-Dodecene, (E)-	0.01	n.d	n.d
	20.471		6-Tridecene, (E)-	n.d	0.02	n.d
	20.536	20.470	6-Tridecene, (Z)-	n.d	0.02	0.01
		20.536	2-Dodecene, 2-methyl-	n.d	n.d	0.01
20.655		20.655	1-Tridecene	0.02	n.d	n.d
20.922	20.940	20.952	Tridecane	3.19	3.45	3.91
	21.565	21.570	trans-Geranic acid methyl ester	n.d	0.05	0.06
		23.128	3-Tetradecene, (E)-	n.d	n.d	0.03
23.128		23.342	7-Tetradecene, (Z)-	0.03	n.d	0.04
	23.342		5-Tetradecene, (E)-	n.d	0.04	n.d
23.342			3-Tetradecene, (Z)-	0.04	n.d	n.d
25.441			1-Octadecyne	0.02	n.d	n.d
23.592	23.592	23.592	Tetradecane	3.14	3.19	3.24
	25.709		9-Eicosene, (E)-	n.d	5.12	n.d
25.816	25.828	25.721	1-Tridecene	0.83	0.96	5.48
26.547	26.607	26.636	Pentadecane	36.2	36.15	37.52
		28.081	(2E,6E)-3,7,11-Trimethyldodeca-2,6,10-trien-1-yl decanoate	n.d	n.d	0.01
28.551	28.563		3-Hexadecene, (Z)-	0.14	0.17	n.d
28.652	28.949	28.658	7-Hexadecene, (Z)-	0.17	0.23	0.38
29.163	29.175	29.175	Hexadecane	0.96	1.08	0.99
31.084			Z-1,6-Tridecadiene	0.34	n.d	n.d
	31.090		6,9-Heptadecadiene	n.d	0.54	n.d
		31.096	Linoelaidic acid	n.d	n.d	0.48
31.429	31.465	31.416	8-Heptadecene	8.85	9.53	9.78
31.869	31.869	31.869	Heptadecene	0.84	1.02	1.08
32.249	32.249	32.249	Pentadecanal-	0.04	0.08	0.10
32.446	32.452	32.457	Z,Z-10,12-Hexadecadienal	0.03	0.02	0.03
		33.278	E,E−10,12-Hexadecadienal	n.d	n.d	0.02
		33.718	9-Octadecene, (E)-	n.d	n.d	0.01
	33.718		3-Octadecene, (E)-	n.d	0.01	n.d
34.212		34.217	cis-9-Hexadecenal	0.24	n.d	0.35
	34.212		7-Hexadecenal, (Z)-	n.d	0.21	n.d
	34.408		13-Octadecenal, (Z)-	n.d	0.03	n.d
34.408		34.408	cis-11-Hexadecenal	0.05	n.d	0.06
	34.699	34.729	Hexadecanal	n.d	0.94	1.89
34.699			Tetradecanal	1.21	n.d	n.d
	35.942		9-Nonadecene	n.d	0.02	n.d
35.942	35.942	36.031	Z-5-Nonadecene	0.02	0.02	0.03
		36.108	Cyclotetradecane	n.d	n.d	0.02
36.495	36.501	36.501	Nonadecane	0.21	0.32	0.32
	38.564		Eicosane	n.d	0.05	n.d
38.594			9-Octadecenal, (Z)-	0.06	n.d	n.d
		38.594	13-Octadecenal, (Z)-	n.d	n.d	0.09
40.491	40.491	40.491	Heneicosane	0.06	0.09	0.07
43.606	43.606	43.606	9-Tricosene, (Z)-	0.02	0.01	0.04
	44.052		Eicosane, 7-hexyl-	n.d	0.01	n.d

**Table 2:** Retention times, names of the identified compounds, and relative peak areas (%) in the GC chromatogram from coffee flower at 2000–2500 mm/year annual rainfall.

Retention Time (min.)			Compounds	Relative Peak Area (%)		
1	2	3		1	2	3
		2.817	Cyclobutanol	n.d	0.27	n.d
3.245	3.245	3.245	Butanenitrile, 2-methyl-	8.76	6.74	6.15
7.663			2-Hexanone, 4-methyl-	0.11	n.d	n.d
7.924			Pentane, 1-propoxy-	1.68	n.d	n.d
7.930			1,6-Heptadien-4-ol	n.d	1.21	n.d
11.272	11.278	11.284	β-myrcene	1.01	0.63	0.65
11.599			7H-Dibenzo[b,g]carbazole, 7-methyl	0.65	n.d	n.d
12.562	12.568	12.574	D-Limonene	0.36	0.24	0.24
12.854	12.842	12.842	Benzyl alcohol	0.58	0.51	0.55
13.222	13.228	13.222	β-Ocimene	1.05	0.44	0.54
14.037			2-Furanmethanol, 5-ethenyltetrahydro-α,α,5-trimethyl-, cis-	0.12	n.d	n.d
14.037	14.043	14.560	Ethyl 2-(5-methyl-5-vinyltetrahydrofuran-2-yl)propan-2-yl carbonate	0.12	0.14	0.21
14.56		14.037	trans-Linalool oxide (furanoid)	0.24	n.d	0.16
14.822		14.822	Benzoic acid, methyl ester	0.22	n.d	0.23
	14.834		3-Methyl-2-(2-methyl-2-butenyl)-furan	n.d	0.12	n.d
14.965	14.965	14.965	Linalool	2.49	1.20	1.41
15.886	15.886	15.886	2,4,6-Octatriene, 2,6-dimethyl-, (E,Z)-	0.09	0.04	0.04
		17.301	(3R,6S)-2,2,6-Trimethyl-6-vinyltetrahydro-2H-pyran-3-ol	n.d	0.02	n.d
17.527	17.533		3,6-Octadienal, 3,7-dimethyl-	0.03	0.03	n.d
17.527			Isogeranial	n.d	n.d	0.03
17.813	17.813	17.813	α-Terpineol	0.02	0.02	0.01
17.902	17.914	17.908	Methyl salicylate	0.03	0.01	0.02
17.997	18.003	17.997	Dodecane	0.08	0.08	0.08
18.651	18.651	18.657	6-Octen-1-ol, 7-methyl-3-methylene	0.03	0.03	0.03
18.972	18.978	18.972	2,6-Octadien-1-ol, 3,7-dimethyl-,(Z)-	3.9	3.24	3.27
	19.061	19.055	3,6-Octadien-1-ol, 3,7-dimethyl-, (Z)-	n.d	0.13	0.14
19.269	19.269	19.270	2,6-Octadienal, 3,7-dimethyl-, (Z)	0.25	0.21	0.21
19.713	19.721	19.710	2,6-Octadien-1-ol, 3,7-dimethyl-, (E)-	2.93	2.31	2.25
20.114	20.114	20.108	2,6-Octadienal, 3,7-dimethyl-, (E)	0.27	0.22	0.21
		20.465	6-Tridecene, (Z)-	n.d	n.d	0.02
20.464			6-Tridecene, (E)-	0.02	n.d	n.d
	20.471		Cyclopentane, hexyl-	n.d	0.02	n.d
20.530			7-Tetradecene, (E)-	0.02	n.d	n.d
		20.536	2-Tridecene, (Z)-	n.d	n.d	0.02
	20.536		4-Tridecene, (Z)-	n.d	0.02	n.d
20.928	20.940	20.940	Tridecane	3.96	3.96	4.42
		23.343	5-Tetradecene, (E)-	n.d	n.d	0.06
23.122	23.122		3-Tetradecene, (E)-	0.06	0.06	n.d
23.342	23.342	23.123	7-Tetradecene, (Z)-	0.06	0.06	0.06
23.592	23.604	23.604	Tetradecane	3.58	3.43	3.61
25.691			n-Tridecan-1-ol	5.19	n.d	n.d
	25.715		1-Hexadecanol	n.d	5.49	n.d
25.816	25.834	25.715	1-Tridecene	0.09	1.47	6.47
26.482	26.553	26.553	Pentadecane	30.35	30.49	35.15
	28.557		6-Tetradecene, (Z)-	n.d	0.12	n.d
28.545	28.646	28.646	7-Hexadecene, (Z)-	0.22	0.23	0.25
28.937		28.557	3-Hexadecene, (Z)-	0.01	n.d	0.12
	28.944		Z-8-Hexadecene	n.d	0.01	n.d
29.151	29.164	29.164	Hexadecane	0.91	0.90	0.93
31.072	31.078	31.084	6,9-Heptadecadiene	0.32	0.31	0.36
31.405	31.441	31.441	8-Heptadecene	8.07	0.37	9.42
31.863	31.875	31.875	Heptadecene	1.08	1.14	1.26
29.538	32.244	32.244	Pentadecanal-	0.03	0.03	0.05
32.466			E,E−10,12-Hexadecadienal	0.03	n.d	n.d
33.272	33.272	33.272	Z,Z-10,12-Hexadecadienal	0.03	0.01	0.02
	34.402	34.212	cis-9-Hexadecenal	n.d	0.02	0.16
	34.212		E−11-Hexadecenal	n.d	0.13	n.d
34.259			Octadecane	0.06	n.d	n.d
		34.402	13-Octadecenal, (Z)-	n.d	n.d	0.02
	34.687		Hexadecanal	n.d	0.61	n.d
34.675	29.532	34.687	Tetradecanal	0.23	0.12	0.65
36.031	35.936	36.025	Z-5-Nonadecene	0.02	0.01	0.02
		35.94	9-Nonadecene	n.d	n.d	0.02
36.495	36.495	36.495	Nonadecane	0.27	0.23	0.30
38.552	38.558	38.558	Eicosane	0.01	0.02	0.04
40.491	40.491	40.492	Heneicosane	0.05	0.06	0.08
43.606	43.606		9-Tricosene, (Z)-	0.01	0.01	n.d
		43.606	11-Tricosene	n.d	n.d	0.01

**Table 3:** Retention times, names of the identified compounds, and relative peak areas (%) in the GC chromatogram from coffee flower at 2500–3000 mm/year annual rainfall.

Retention Time (min.)			Compounds	Relative Peak Area (%)		
1	2	3		1	2	3
		1.800	Ethanol	n.d	n.d	5.19
3.269	3.263	3.263	Butanenitrile, 2-methyl-	11.8	12.63	11.59
4.309	4.315		2-Propanone, O-methyloxime	8.65	6.82	n.d
		6.581	Butanoic acid, 3-methyl-, ethyl ester	n.d	n.d	0.36
	6.604		Pentanoic acid, ethyl ester	n.d	0.16	n.d
7.936			Pentane, 1-propoxy-	1.65	n.d	n.d
		7.948	Oxalic acid, pentyl propyl ester	n.d	n.d	1.56
		9.488	2-Butenoic acid, 2-methyl-, ethyl ester	n.d	n.d	0.02
	10.220		2-Phenylindolizine	n.d	0.02	n.d
		10.220	Carbamic acid, (3-ethylphenyl)-, ethyl ester	n.d	n.d	0.03
10.588		10.576	1-Heptanol	0.03	n.d	0.04
11.260	11.266	11.266	β-myrcene	0.41	0.11	0.30
12.562	12.580	12.574	D-Limonene	0.16	0.12	0.13
12.854			Benzyl alcohol	0.19	n.d	n.d
	12.854		E,Z-3-Ethylidenecyclohexene	n.d	0.11	n.d
		12.860	Spiro[2.4]heptane, 4-methylene-	n.d	n.d	0.11
13.222	13.210	13.288	β-Ocimene	0.21	0.21	0.15
	14.025		2-Furanmethanol, 5-ethenyltetrahydro-α,α,5-trimethyl-, cis-	n.d	0.25	n.d
		14.031	trans-Linalool oxide (furanoid)	n.d	n.d	0.29
14.548	14.548	14.548	Ethyl 2-(5-methyl-5-vinyltetrahydrofuran-2-yl)propan-2-yl carbonate	0.58	0.40	0.48
14.828	14.828	14.828	3-Methyl-2-(2-methyl-2-butenyl)-furan	0.21	0.20	0.25
14.952	14.953	14.958	Furan, 3-(4-methyl-3-pentenyl)-	0.98	0.82	1.21
15.178	15.184	15.184	Butanoic acid, 3-methyl-, 2-methylbutyl ester	0.06	0.09	0.07
	15.541		5H-Naphtho[2,3-c]carbazole	n.d	0.02	n.d
15.874	15.874	15.880	2,4,6-Octatriene, 2,6-dimethyl-, (E,Z)-	0.06	0.04	0.05
16.344			1,3-Cyclopentadiene, 1,2,3,4,5-pentamethyl	0.02	n.d	n.d
		16.350	6-Octenal, 7-methyl-3-methylene-	n.d	n.d	0.02
		16.974	Isogeranial	n.d	n.d	0.05
	17.164	17.170	Benzoic acid, ethyl ester	n.d	0.02	0.05
17.283	17.283	17.283	(3R,6S)-2,2,6-Trimethyl-6-vinyltetrahydro-2H-pyran-3-ol	0.06	0.04	0.06
	17.521		1H-Indene, 1-methylene-	n.d	0.03	n.d
17.521			Naphthalene	0.04	n.d	n.d
	17.795		Terpineol	n.d	0.01	n.d
17.795		17.795	L-α-Terpineol	0.02	n.d	0.02
17.991	17.991	17.991	Dodecane	0.09	0.09	0.09
18.354			Hexanoic acid, 2-methylbutyl ester	0.01	n.d	n.d
18.621	18.621	18.621	6-Octen-1-ol, 7-methyl-3-methylene	0.01	0.01	0.01
	18.746	18.640	Neryl nitrile	n.d	0.01	0.01
18.907	18.889	18.895	2,6-Octadien-1-ol, 3,7-dimethyl-,(Z)-	1.11	0.57	0.73
19.031			Cyclopentanol, 1-(methylenecyclopropyl)	0.08	n.d	n.d
		19.031	3,6-Octadien-1-ol, 3,7-dimethyl-, (Z)-	n.d	n.d	0.08
19.245	19.246	19.246	2,6-Octadienal, 3,7-dimethyl-, (Z)	0.12	0.06	0.18
19.656	19.638	19.650	2,6-Octadien-1-ol, 3,7-dimethyl-, (E)-	1.01	0.50	0.68
20.090	20.090	20.090	2,6-Octadienal, 3,7-dimethyl-, (E)	0.13	0.08	0.21
	20.310		Eicosane, 1-iodo-	n.d	0.03	n.d
		20.310	Tetradecane, 5-methyl-	n.d	n.d	0.03
20.310			Octane, 5-ethyl-2-methyl-	0.03	n.d	n.d
		20.453	5-Tridecene, (E)-	n.d	n.d	0.01
20.530	20.524		6-Tridecene, (E)-	0.03	0.02	n.d
		20.530	3-Tridecene, (E)-	n.d	n.d	0.02
20.922	20.928	20.928	Tridecane	3.93	4.15	3.97
	21.559		trans-Geranic acid methyl ester	n.d	0.09	n.d
21.832			Nonadecane	0.02	n.d	n.d
	21.838		Hexane, 3,3-dimethyl-	n.d	0.02	n.d
		21.838	3-Ethyl-3-methylheptane	n.d	n.d	0.01
	22.028		1-Hexanol, 5-methyl-2-(1-methylethyl)-	n.d	0.01	n.d
	22.094		Octane, 5-ethyl-2-methyl-	n.d	0.01	n.d
22.094			Nonane, 1-iodo-	0.01	n.d	n.d
23.087	28.545		7-Tetradecene	0.04	0.13	n.d
		23.087	7-Tetradecene, (Z)-	n.d	n.d	0.04
	23.104	28.539	5-Tetradecene, (E)-	n.d	0.05	0.11
	23.122		3-Tetradecene, (E)-	n.d	0.04	n.d
23.330		23.330	3-Tetradecene, (Z)-	0.03	n.d	0.04
23.568	23.580	23.580	Tetradecane	2.17	2.92	2.72
25.435			11,14-Eicosadienoic acid, methyl ester	0.01	n.d	n.d
25.679			Cyclopentadecane	4.43	n.d	n.d
		25.679	1-Hexadecanol	n.d	n.d	4.42
25.810	25.685	25.810	1-Tridecene	1.08	1.21	1.04
26.482	26.500	26.494	Pentadecane	27.46	29.51	27.82
26.696			1-Pentadecene	0.02	n.d	n.d
		27.403	Hexadecane, 1-iodo-	n.d	n.d	0.01
	27.409		Docosane, 1-iodo-	n.d	0.01	n.d
27.843			4,5-Nonadiene	0.01	n.d	n.d
28.045			Supraene	0.02	n.d	n.d
		28.046	2-Octene, 2-methyl-6-methylene-	n.d	n.d	0.01
	28.052		1,6,10,14-Hexadecatetraen-3-ol, 3,7,11,15-tetramethyl-, (E,E)-	n.d	0.01	n.d
	28.295		Chiloscyphone	n.d	0.01	n.d
28.295			Acetic acid, 1,3,7-trimethylocta-2,6-dienyl ester	0.01	n.d	n.d
28.545	23.330		7-Tetradecene, (E)-	0.01	0.04	n.d
28.928	28.634	28.628	7-Hexadecene, (Z)-	n.d	0.22	0.19
28.628	28.926		3-Hexadecene, (Z)-	0.18	0.01	n.d
29.140	29.146	29.146	Hexadecane	0.77	0.88	0.87
29.520	29.532	29.526	Pentadecanal-	0.04	0.02	0.03
31.066	31.066		6,9-Heptadecadiene	0.21	0.02	n.d
		31.066	9-Tetradecen-1-ol, acetate, (E)-	n.d	n.d	0.21
		32.246	Z-1,6-Tridecadiene	n.d	n.d	0.01
31.435	31.423	31.447	8-Heptadecene	9.91	8.95	10.08
31.875	31.869	31.881	Heptadecene	1.59	1.37	1.71
	32.089		Cyclopentane, pentyl-	n.d	0.01	n.d
		32.095	Cyclopentane, nonyl-	n.d	n.d	0.01
32.089			1-Octadecene	0.01	n.d	n.d
	32.249		Cyclotridecane	n.d	0.01	n.d
32.237			Octadecanal	0.02	n.d	n.d
33.260	33.266	33.266	Z,Z-10,12-Hexadecadienal	0.02	0.01	0.01
		33.712	E−7-Octadecene	n.d	n.d	0.01
33.712			9-Octadecene, (E)-	0.01	0.01	n.d
34.247	34.253	34.253	Octadecane	0.10	0.07	0.09
		34.390	E−11-Hexadecenal	0.10	n.d	n.d
	34.396		13-Tetradecenal	n.d	0.01	n.d
		34.396	13-Octadecenal, (Z)-	n.d	n.d	0.01
34.669	34.669		Hexadecanal	0.56	0.30	n.d
		34.669	Tetradecanal	n.d	n.d	0.43
35.930	35.930	35.930	Z-5-Nonadecene	0.02	0.02	0.02
	36.019	36.019	1-Heptadecene	n.d	0.03	0.03
36.483	36.489	36.483	Nonadecane	0.35	0.3	0.38
38.546	38.546	38.546	Eicosane	0.03	0.02	0.02
40.478	40.479	40.479	Heneicosane	0.08	0.06	0.06
43.594	43.600	43.600	9-Tricosene, (Z)-	0.00	0.00	0.00

**Table 4:** Retention times, names of the identified compounds, and relative peak areas (%) in the GC chromatogram from coffee flower at 3000–3500 mm/year annual rainfall.

Retention Time (min.)			Compounds	Relative Peak Area (%)		
1	2	3		1	2	3
2.703		2.703	1-Butanamine, N,3-dimethyl-	0.33	n.d	0.28
	2.858		Cyclobutanol	n.d	0.27	n.d
3.245	3.233	3.245	Butanenitrile, 2-methyl-	6.23	6.01	4.88
4.309			1,3-Dioxolane, 2-ethenyl-4-methyl-	6.42	n.d	n.d
		4.321	Tetrazole, 1-(1,3-dioxolan-4-ylmethyl)-	n.d	n.d	4.95
7.930			Oxalic acid, pentyl propyl ester	1.02	n.d	n.d
		7.930	Pentane, 1-propoxy-	n.d	n.d	0.42
		10.267	Benzaldehyde	n.d	n.d	0.07
	11.123	11.123	5-Hepten-2-one, 6-methyl-	0.46	0.90	n.d
11.245		11.260	β-myrcene	1.35	n.d	2.20
12.538	12.562	12.556	D-Limonene	0.37	0.11	0.40
12.859		12.854	Benzyl alcohol	0.49	n.d	1.10
	12.871		trans-β-Ocimene	n.d	0.06	n.d
13.222	13.216	13.234	β-Ocimene	1.45	0.42	1.40
	14.037	14.043	Ethyl 2-(5-methyl-5-vinyltetrahydrofuran-2-yl)propan-2-yl carbonate	n.d	0.03	0.06
14.037	14.560		trans-Linalool oxide (furanoid)	0.06	0.08	n.d
		14.560	2-Carene	n.d	n.d	0.22
14.828	14.834		3-Methyl-2-(2-methyl-2-butenyl)-furan	0.15	0.05	n.d
14.958	14.952	14.964	Linalool	1.26	0.40	1.59
15.886	15.886	15.892	2,4,6-Octatriene, 2,6-dimethyl-, (E,Z)-	0.19	0.05	0.23
		16.284	2,4,6-Octatriene, 3,4-dimethyl-, (E,Z)-	n.d	n.d	0.08
		16.998	cis-Verbenol	n.d	n.d	0.02
17.301			(3R,6S)-2,2,6-Trimethyl-6-vinyltetrahydro-2H-pyran-3-ol	0.02	n.d	n.d
		17.533	3,6-Octadienal, 3,7-dimethyl-	n.d	n.d	0.04
17.527			Isoneral	0.02	n.d	n.d
17.807			L-α-Terpineol	0.03	n.d	n.d
		17.824	α-Terpineol	n.d	n.d	0.06
18.003	18.003	18.003	Dodecane	0.07	0.07	0.06
18.651		18.686	6-Octen-1-ol, 7-methyl-3-methylene	0.03	n.d	0.06
18.984	18.907	19.019	2,6-Octadien-1-ol, 3,7-dimethyl-,(Z)-	3.99	0.79	6.01
19.269	19.257	19.293	2,6-Octadienal, 3,7-dimethyl-, (Z)	0.2	0.06	0.35
19.727	19.656	19.775	2,6-Octadien-1-ol, 3,7-dimethyl-, (E)-	2.97	0.78	5.08
20.114	20.102	20.137	2,6-Octadienal, 3,7-dimethyl-, (E)	0.22	0.07	0.40
		20.387	2,6-Octadienoic acid, 3,7-dimethyl -, methyl ester	n.d	n.d	0.09
	20.464		4-Nonene, 5-butyl-	n.d	0.01	n.d
28.557	20.530		7-Tetradecene, (E)-	0.11	0.02	n.d
20.940	20.946	20.934	Tridecane	4.14	4.83	3.88
21.564			trans-Geranic acid methyl ester	0.08	n.d	n.d
		23.128	7-Tetradecene	n.d	n.d	0.03
23.336	23.122		3-Tetradecene, (E)-	0.05	0.04	n.d
23.604	23.628	23.598	Tetradecane	3.84	5.44	3.43
	25.703		1-Hexadecanol	n.d	5.35	n.d
25.679	25.822	25.673	1-Tridecene	3.78	1.14	0.71
26.553	26.624	26.547	Pentadecane	34.27	41.96	32.6
		28.063	1,6,10,14-Hexadecatetraen-3-ol, 3,7,11,15-tetramethyl-, (E,E)-	n.d	n.d	0.01
	28.652		7-Hexadecene, (Z)-	n.d	0.2	n.d
28.640			3-Hexadecene, (Z)-	0.18	n.d	n.d
		28.634	Z-8-Hexadecene	n.d	n.d	0.30
29.163	29.175	29.163	Hexadecane	1.09	1.24	1.03
	31.084	31.078	6,9-Heptadecadiene	n.d	0.47	0.31
31.078			Z,Z-10,12-Hexadecadienal	0.36	n.d	n.d
31.417	31.417	31.405	8-Heptadecene	7.53	7.12	6.68
31.869	31.875	31.863	Heptadecene	1.13	1.28	0.94
	32.243	29.538	Pentadecanal-	n.d	0.05	0.04
		32.255	1,15-Pentadecanediol	n.d	n.d	0.02
32.440			3,4-Octadiene, 7-methyl-	0.01	n.d	n.d
33.272	32.440	32.445	Z,Z-10,12-Hexadecadienal	0.02	0.02	0.02
	34.211		cis-9-Hexadecenal	n.d	0.12	n.d
34.259		34.253	Octadecane	0.07	n.d	0.06
	34.402		13-Octadecenal, (Z)-	n.d	0.02	n.d
34.675			Hexadecanal	0.36	n.d	n.d
31.249	34.681	34.675	Tetradecanal	0.02	0.65	0.41
35.936	35.936		Z-5-Nonadecene	0.02	0.03	n.d
		35.936	9-Nonadecene	n.d	n.d	0.22
36.495	36.495		Nonadecane	0.31	0.32	n.d
38.552	38.552	38.558	Eicosane	0.02	0.02	0.03
40.484	40.484	40.484	Heneicosane	0.06	0.04	0.05
	43.606	43.606	9-Tricosene, (Z)-	n.d	0.01	0.01

## 2 Experimental design, materials, and methods

The sample of coffee flower was collected from four locations at different annual rainfall (1500–2000, 2000–2500, 2500–3000, 3000–3500 mm/year). At each location, the coffee flowers were selected and only fresh anthesis flowers were used for this analysis following [1], [2], [3] method. From every location, ten fresh flowers (ca. 1.3 g) were placed respectively in 22 mL solid phase micro extraction (SPME) clear glass vial (Supelco Co., Bellefonte, PA, USA) with PTFE/silicone septa and will be identified using gas chromatography - mass spectrometry (GCMS) after 24hr waiting period. Three set of samples were taken from each location. Analysis of volatile compounds of coffee flowers was conducted in ICRR flavor laboratory (Indonesian Centre for Rice Research) West Java, Indonesia. The flowers were extracted using the procedure of [2] with some modifications. All samples were extracted at 40 °C for 45 minutes. With splitless mode, SPME was injected into a gas chromatograph (Agilent 7890A) at 250 °C for 5 minutes. The oven temperature initially was set at 50 °C held for 5 minutes and then increased to 150 °C at the rate of 5 °C/min for 2 minutes and then increased to 250 °C at the rate of 5 °C/min for 5 minutes. The volatile compounds were identified based on their retention times in gas chromatograph equipped with mass spectrometer. HP-5MS (30 m × 250 μm x 0.25 μm) column was used for the separation. Gas carrier was helium 0.8 ml/min. The relative amounts of volatile compounds from each location were determined by comparing spectra of each compound with library NIST14.

## References

[1] Emura M., Nohara I., Toyoda T., Kanisawa T. (1997). The volatile constituents of the coffee flower *(Coffea arabica L.)*. Flavour Fragrance J..

[2] Hetelyi E.B., Szarka S., Lemberkovics E., Szoke E. (2010). SPME-GC/MS Identification of aroma compounds in rose flowers. Acta Agron. Hung..

[3] Stashenko E.E., Martínez J.R., Cárdenas-Vargas S., Saavedra-Barrera R., Durán D.C. (2013). GC–MS study of compounds isolated from *Coffea arabica* flowers by different extraction techniques. J. Sep. Sci..

